# A new unique recombinant HIV-1 revealed in Belarus

**DOI:** 10.1186/1742-4690-9-S1-P32

**Published:** 2012-05-25

**Authors:** VF Eremin, EL Gasich, SV Sasinovich

**Affiliations:** 1 Briem, Minsk, Belarus

## Material and methods

Blood plasma, EIA, western blot, RT-PCR, sequencing, SeqScape, BioEdit, Mega4.1, statistica 6.0, software.

## Results

In April 2010 we have performed resistance tests of plasma sample obtained from patient Mos, 6 years old girl, born from HIV-infected mother. The phylogenetic analysis of the DNA fragment of patient Mos had shown that sample has been clustered with HIV-1 subtype A on gene pol, but was different from other analyzed samples, subtype A consensus IDU-A and reference sequences (AF004885). The Mos isolate is the most similar to AF413987 from Ukraine (subtype A) the p-distance was 0.066. The comparison of sequences from gag gene p17/p24 region of Mos isolate with reference sequences HIV-1 of subtype A demonstrates that average p-distance was 0.129, and with reference sequences of subtype B was 0.075. Average p-distance on gag gene (the Mos isolate) with CRF03_AB (AF414006.1, Belarus and AF193276.1 CRF03_AB KAL153) was 0.121 if compared with 0.013 p-distance between reference sequences. The analysis of Mos isolate sequences on V3 loop gp120 gene env region HIV-1 has shown that average p-distance with reference isolates subtype B was 0.323, and with A subtype was 0.155. Average p-distance sequence of Mos isolate with reference isolates AF414006.1 and AF193276.1 (CRF-03_AB) was 0.308.

## Conclusion

Thus, it has been shown that Mos isolate is a unique recombinant form, but differs in genome structure from the one described earlier CRF03_ AB (AgagBpolBenv). The new recombinant HIV-1 has the following structure: BgagApolAenv. Sequences of new HIV-1 unique recombinant in gag, pol and env genes were submitted to EMBL/Genbank/DDBJ under accession numbers: FR775442.1, FN995656.1, FR775443.1.

